# Cardiac Tamponade After Late Central Venous Catheter Dislodgement in Two Pediatric Patients—A Rare but Potentially Fatal Complication

**DOI:** 10.3390/children13050689

**Published:** 2026-05-18

**Authors:** Zdravko Ivanov, Ivelina Neycheva, Zeyra Halil, Georgi Bukov, Fani Galabova, Sadika Ali, Atanas Kerezov, Ivanka Paskaleva, Ivan Yankov

**Affiliations:** 1 Pediatrics Clinic, St. George University Hospital, 4002 Plovdiv, Bulgariaivanka.paskaleva@mu-plovdiv.bg (I.P.);; 2 Department of Pediatrics, Medical University of Plovdiv, 4002 Plovdiv, Bulgaria

**Keywords:** central venous catheter, cardiac tamponade, pericardiocentesis, echocardiography, children

## Abstract

**Highlights:**

**What are the main findings?**

- Late displacement of a central venous catheter (CVC) may result in cardiac tamponade and sudden cardiovascular collapse in pediatric patients.
- CVC-related cardiac tamponade, although rare, represents a rapidly fatal complication requiring immediate clinical recognition.

**What is the implication of the main finding?**

- Cardiac tamponade should be systematically considered in any child with a CVC presenting with unexplained hemodynamic deterioration.
- Strict verification of catheter tip position, routine assessment of blood aspiration, and secure catheter fixation are essential preventive measures in clinical practice.

**Abstract:**

**Background:** Cardiac tamponade (CT) is a rare but life-threatening medical emergency caused by fluid accumulation in the pericardial sac, impairing cardiac filling and reducing output. More than 20% of CT cases are iatrogenic. CT is a recognized complication of central venous catheter (CVC) placement, with mortality rates in pediatric patients reported to reach 50%. Clinical presentation is often nonspecific, and echocardiography remains the diagnostic gold standard. **Case report:** We present two pediatric cases of CT due to late CVC migration, managed in the pediatric intensive care unit (PICU). The first case involved a 25-day-old neonate with short bowel syndrome who received prolonged parenteral nutrition via CVC. Four days after catheter insertion, the patient developed sudden cardiocirculatory collapse. The second case featured a 2-year-old child with Leigh syndrome who required mechanical ventilation and multimodal pharmacological therapy. Six days after CVC placement, the patient developed acute hemodynamic deterioration. In both cases, echocardiography confirmed CT, while chest radiography suggested intracardiac positioning of the catheter tip. **Management and outcome:** Emergency pericardiocentesis and advanced cardiopulmonary resuscitation were performed. Despite transient hemodynamic stabilization, both patients developed multiorgan failure with fatal outcomes. **Conclusions:** CT is a critical complication in pediatric patients with CVCs. Accurate verification of catheter tip position is essential, and intracardiac placement should be avoided. Any sudden clinical deterioration in a patient with a CVC should raise suspicion of late catheter migration and requires immediate life-saving intervention.

## 1. Introduction

Cardiac tamponade (CT) is a medical emergency characterized by the accumulation of fluid within the pericardial sac, leading to compression of the cardiac chambers, impaired ventricular filling, reduced cardiac output, and ultimately cardiogenic shock. More than 20% of cases are iatrogenic and occur secondary to surgical procedures, invasive interventions, or the administration of certain medications [1]. Central venous catheter (CVC) placement is a well-documented cause of cardiac tamponade. In pediatric patients, the reported incidence ranges from 1% to 3%, with mortality rates between 30% and 50%, according to different studies [2]. Nowadays CVCs are widely used for the administration of blood products, hyperosmolar fluids, vasoactive medications, chemotherapy, parenteral nutrition, as well as for hemodynamic monitoring and insertion of temporary pacemakers. Consequently, the increasing use and prolonged placement of CVCs have been associated with a higher incidence of iatrogenic complications [3].

In this article, we present two cases of pediatric patients treated in the pediatric intensive care unit (PICU) who developed cardiac tamponade following CVC placement, both with fatal outcomes. We also discuss potential risk factors for this complication in the pediatric population, preventive strategies, and therapeutic interventions to be considered when CT is suspected.

## 2. Case Report

**Figure 1:**
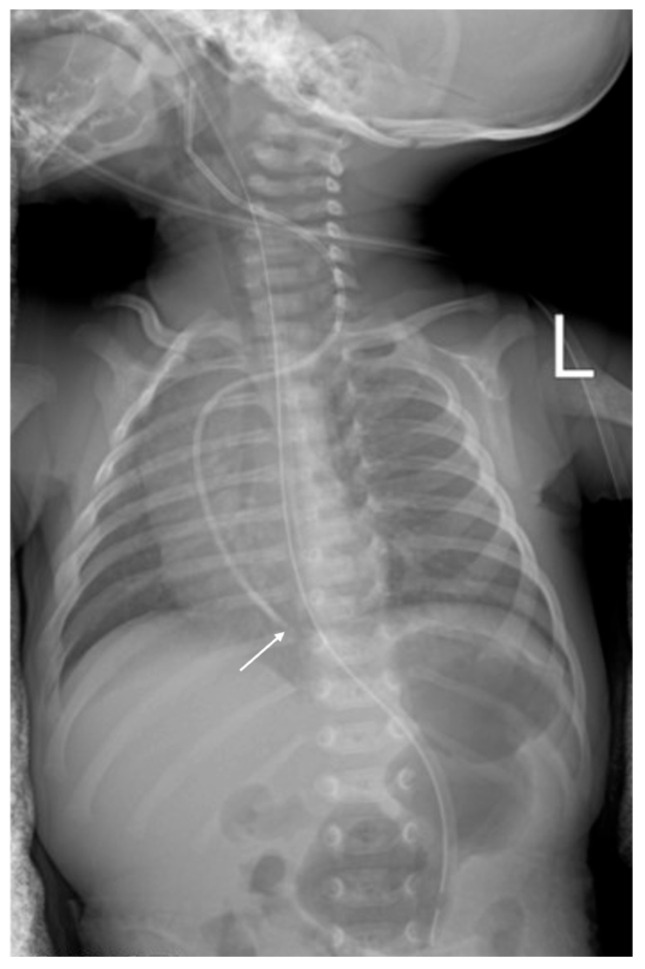
Chest radiography of Patient 1 right after CVC placement. Catheter tip located intracardially (white arrow). L: left.

**Figure 2:**
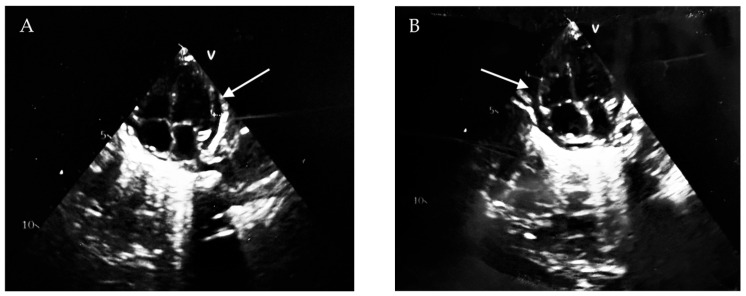
(**A**,**B**) Echocardiography of Patient 1 after pericardiocentesis—a residual pericardial effusion is visualized, mainly around the right ventricle (white arrows).

**Case 1.** A 25-day-old full-term neonate was born to a primigravida, primiparous mother following an unmonitored pregnancy and uncomplicated vaginal delivery, with normal Apgar scores at 1 and 5 min. Shortly after birth, high anorectal atresia without fistula was diagnosed, and the patient was referred to the Department of Pediatric Surgery for corrective intervention. Postoperatively, the neonate developed necrotizing enterocolitis, which necessitated extensive intestinal resection with subsequent ileostomy formation, ultimately resulting in short bowel syndrome. The patient was then transferred to the pediatric intensive care unit (PICU) for further management. At admission, the patient weighed 2550 g and measured 47 cm in length. Physical examination revealed a postoperative scar on the anterior abdominal wall and macerated peristomal skin surrounding the ileostomy site. Laboratory investigations revealed a hemoglobin level of 98 g/L, hematocrit of 0.31, platelet count of 354 × 10^9^/L, and a prothrombin activity of 63%. Owing to inadequate physical growth and nutritional status, the patient required partial parenteral nutrition in combination with enteral formula feeding administered via a nasogastric tube. A dual-lumen 4 Fr, 13 cm polyurethane central venous catheter with a soft tip (Certofix^®^ Duo Paed S413, B. Braun, Melsungen, Germany) was inserted in the left internal jugular vein via the Seldinger technique using a flexible J-tip guidewire. Ultrasound guidance was not used during catheter insertion. Catheter patency was confirmed by free blood aspiration and saline flushing of all lumens. Post-procedural chest radiography showed that the catheter tip was situated 4.5 cm below the carina, indicating intracardiac placement. However, this finding was overlooked, and the catheter was not repositioned (Figure 1). Hyperosmolar parenteral nutrition was administered daily through the CVC, with an initial total volume of 210 mL/day that was subsequently increased to 300 mL/day due to reduced enteral feeding tolerance. The infusion rate ranged from 10 to 15 mL/h. On day 4 after CVC placement, the patient developed rapidly progressive hemodynamic instability followed by cardiopulmonary arrest. Approximately four hours before the event, parenteral nutrition had been temporarily discontinued and replaced with crystalloid infusions. Advanced life support was initiated immediately and included mechanical ventilation, cardiopulmonary resuscitation, and pharmacologic resuscitative therapy. Despite these interventions, the patient remained hemodynamically unstable, prompting transthoracic echocardiography, which confirmed CT. Emergency pericardiocentesis was performed, yielding an immediate drainage of 40 mL of serous fluid and resulting in transient hemodynamic improvement (Figure 2A,B). However, during the following days, the patient developed multiorgan failure characterized by lactic acidosis, acute kidney injury, impaired consciousness, and treatment-refractory seizures. Three days after the acute event, wide-complex tachycardia was documented. Despite initiation of antiarrhythmic therapy, the arrhythmia progressed to cardiac arrest, resulting in death.

**Figure 3:**
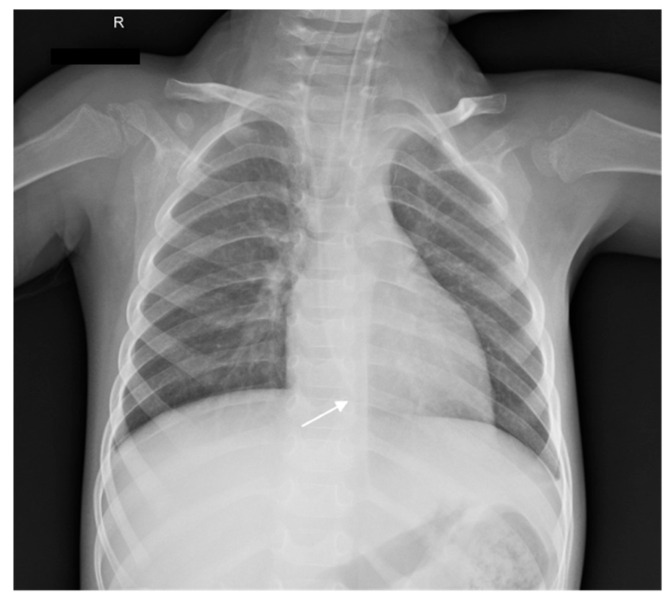
Chest radiography of Patient 2 upon admission showing the intracardiac position of the catheter tip (white arrow). R: right.

**Figure 4:**
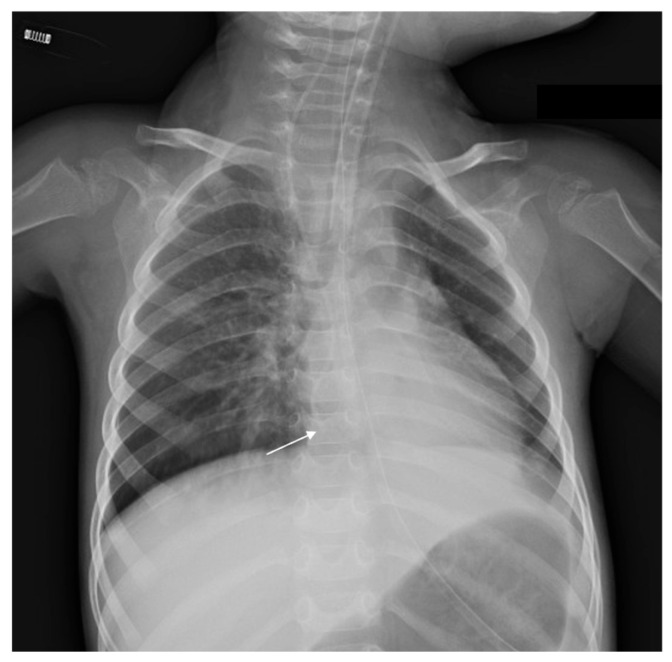
Chest radiography of Patient 2—on day 3 of hospitalization, S-shaped folding of the catheter tip can be visualized, raising the suspicion of dislocation (white arrow).

**Case 2.** A 2-year-old boy with Leigh syndrome was born at term following an uncomplicated pregnancy. The perinatal period was uneventful. His past medical history included a SARS-CoV-2 infection at 7 months of age and an episode of acute gastroenteritis at 2 years of age. He exhibited global developmental delay with subsequent regression, muscle hypotonia, and lactic acidosis, with clinical deterioration during febrile illnesses. The patient was transferred to the PICU due to severe pneumonia requiring continuous mechanical ventilation. One day prior to transfer, a CVC had been placed in the right internal jugular vein at an external facility. On admission, the patient was afebrile and presented with decreased consciousness (Glasgow Coma Scale score: 8) while on mechanical ventilation. Physical and neurological examination revealed subcutaneous hematomas in the inguinal region (likely iatrogenic in origin), severe generalized hypotonia, diminished deep tendon reflexes, and bilateral positive Babinski signs. Correct CVC function was verified by unobstructed infusion flow and appropriate blood aspiration. Chest radiography performed on admission demonstrated that the catheter tip was located 5 cm below the carina, consistent with intracardiac positioning (Figure 3). A follow-up radiograph obtained two days later to assess the progression of pulmonary pathology confirmed persistent intracardiac catheter tip positioning with mild bending of the distal tip. This finding was underestimated, and no corrective measures were undertaken (Figure 4). On day 5 of hospitalization (6 days after CVC insertion), the patient experienced an acute episode of bradycardia progressing to cardiocirculatory arrest. Advanced life support was initiated immediately. The ongoing hemodynamic instability and inadequate response to resuscitative therapy prompted urgent transthoracic echocardiographic evaluation, which confirmed CT. All intravenous infusions were discontinued, and the CVC was removed. Emergency pericardiocentesis was performed, yielding 120 mL of serous fluid and resulting in transient hemodynamic stabilization. Biochemical analysis of the pericardial fluid revealed a leukocyte count of 30 cells/µL, glucose concentration of 17.8 mmol/L, albumin level of 1 g/L, and a specific gravity of 1.006, findings consistent with non-exudative fluid and suggestive of admixture with the administered intravenous solutions. Despite initial improvement, the patient subsequently developed multiorgan failure, severe coma (GCS score: 3), brainstem areflexia, hypotension, and hypothermia. Twelve days later, the patient passed away (Table 1).

## 3. Discussion

### 3.1. Overview

CT is a well-documented but frequently underrecognized complication of CVC placement, with the first clinical reports dating back to 1958 [4]. It may result from direct mechanical injury during catheter insertion, catheter tip migration, or mechanical and chemical erosion. Direct mechanical injury occurs at the time of insertion due to trauma caused by the guidewire, introducer, or catheter itself to the central venous wall or cardiac chambers and typically presents with acute clinical symptoms [5]. This mechanism is largely dependent on operator experience and catheter properties; stiffer catheters are associated with a higher risk of vascular or cardiac perforation [2]. Mechanical erosion develops when the catheter tip exerts abrasive forces on the constantly moving endocardial surface, leading to thrombus formation, necrosis and eventual perforation [2]. This mechanism is more likely when the catheter tip is positioned intracardially at a more perpendicular angle (>40°) to the vessel or heart wall, particularly in catheters inserted via the left internal jugular or subclavian veins [4]. Chemical erosion occurs when hyperosmolar infusions, such as those used in total parenteral nutrition, induce osmotic injury to the endocardium, potentially resulting in tissue damage and perforation [2].

Depending on the timing of onset, CVC-related CT may be classified as early (occurring within minutes to hours after insertion) or late (developing days to weeks later) [2]. More than one-third of cases occur within the first 24 h, suggesting an intraprocedural origin, while over 80% manifest within the first week following catheter placement. The most commonly affected sites are the right atrium and right ventricle, which together account for approximately 80% of reported perforations [1].

### 3.2. Diagnosis

Early recognition of CT is essential to reduce mortality, as it enables timely initiation of life-saving interventions. Clinical diagnosis is primarily based on symptoms and physical findings, including dyspnea, chest pain or discomfort, tachycardia, tachypnea, and pulsus paradoxus. The classical Beck’s triad—hypotension, jugular venous distension, and muffled heart sounds—is absent in up to 30% of cases [2]. Typical electrocardiographic (ECG) signs are low voltage QRS-complexes and electrical alternans [1]. Chest radiography may demonstrate an increased cardiac silhouette consistent with pericardial effusion [2,6]. Respiratory variability in the pulse-oximetry waveform, considered equivalent of pulsus paradoxus, has also been identified as a sign of hemodynamically significant pericardial effusion and CT [7,8]. Echocardiography is the diagnostic modality of choice and the most sensitive and specific tool for confirming cardiac tamponade. Characteristic findings include diastolic collapse of the right atrium and right ventricle, a dilated inferior vena cava with reduced or absent inspiratory collapse, and the “swinging heart” phenomenon. Doppler echocardiography may additionally demonstrate marked respiratory variation in transvalvular flow velocities [9].

### 3.3. Management

Cardiac tamponade should be suspected in any patient with a central venous catheter who develops sudden tachycardia, hypotension, or acute circulatory collapse. In such cases, all catheter infusions should be stopped immediately. The infusion system should be lowered below the level of the heart, and gentle aspiration of pericardial fluid through the catheter may be attempted. The CVC should then be carefully removed, and urgent echocardiographic evaluation should be performed to confirm the diagnosis. If death is imminent subxiphoid pericardiocentesis has to be attempted [5,6]. Thoracotomy is preserved as a last resort when cardiac perforation is suspected [5].

### 3.4. Measures to Reduce the Risk of CT

CT following CVC placement cannot be completely eliminated in all clinical scenarios; however, several preventive strategies may reduce its incidence and associated mortality. Appropriate catheter selection is essential to minimize procedural risk. Silicone (silastic) catheters are generally preferred over nylon or polyethylene catheters due to their greater flexibility and reduced rigidity. In addition, catheter length should be carefully tailored to the patient, with the aim of avoiding intracardiac placement. To improve placement accuracy, a research group at Texas Children’s Hospital developed a formula to estimate optimal catheter insertion length based on a cohort of 330 pediatric patients undergoing central venous catheterization [10]:

- if the patient’s height is less than 100 cm, the initial length of insertion in cm is (height in cm/10) − 1 cm
- if the patient’s height is more than 100 cm, the formula is (height in cm/10) − 2 cm.

For optimal vascular access, the puncture site should be selected as cranially as possible when performing internal jugular vein cannulation and as laterally as possible when the subclavian vein approach is used [4]. J-tipped guidewires are preferred over sharp or straight guidewires. The guidewire should be advanced gently into the vessel and never forced in the presence of resistance [11]. Whenever feasible, point-of-care ultrasound guidance should be employed to reduce the risk of procedural complications [1].

During insertion, the absence of pulsatile flow and the presence of dark, non-pulsatile blood are typically indicative of venous rather than arterial cannulation. After installation of a CVC, correct tip position should always be confirmed using chest radiography, fluoroscopy, or endocavitary ECG guidance [2].

The optimal position of the catheter tip remains a matter of debate, as different complications may occur at various locations. Available evidence consistently indicates that intracardiac positioning should be avoided to minimize the risk of some adverse effects, including CT [2,4]. Importantly, catheter tips are not fixed and may migrate after insertion, particularly when left internal jugular or subclavian vein access is used [12]. Changes in body position, flexion and extension of the neck, upper limb movements, routine patient handling and even physiological movements of the cardiac and respiratory cycle can all contribute to late catheter dislodgement [1,2]. Anatomical variability in venous structures, as well as short vascular distances additionally increase the risk of late catheter migration, especially in pediatric patients [1,2]. All these factors can potentially transform an initially acceptable paramediastinal position into intracardiac hazard.

Radiographic confirmation of CVC position may be performed using the “Greenhall criterion” according to which the catheter tip should not lie more than 2 cm below an imaginary line connecting the inferior borders of the clavicular heads, or the “right main bronchus (RMB) criterion,” which requires the tip to remain proximal to the angle formed between the RMB and the trachea [4]. The applicability of these criteria in the pediatric population, however, remains uncertain. Currently, there is no evidence that routine radiographic assessment alone prevents delayed catheter-related complications [2,5]. In addition, antero-posterior (AP) chest radiography alone has limited sensitivity for excluding intra-arterial or extravascular malposition [4]. Other limitations of chest radiography include imprecise visualization of catheter borders caused by motion-related image blurring as well as considerable interobserver variability in interpretation [13]. Importantly, inaccurate visualization of the CVC on chest radiography may lead to inappropriate catheter repositioning, potentially compromising effective therapy [14]. Despite these limitations, chest radiography remains an essential initial tool for excluding gross catheter malposition, including intracardiac placement [4]. A multicenter study conducted in intensive care units in the United States reported intracardiac catheter tip location in 47% of patients, with no subsequent adjustment of catheter position after radiographic assessment, suggesting that AP chest radiography is often underappreciated as a tool to evaluate CVC tip position [15]. A reasonable clinical strategy is to confirm CVC placement with chest radiography after insertion and to reassess catheter tip position whenever additional chest imaging is obtained for other indications [1].

Securely anchoring the external portion of the catheter to the skin is essential to minimize the risk of migration. Routine assessment of blood backflow should be performed regularly under strict aseptic conditions [2,4]. Verification of free blood return from all lumens prior to initiating high-volume infusions is critical for ensuring correct catheter function. Finally, high-flow infusions and the administration of hyperosmolar solutions through the distal lumen should be avoided whenever possible, as they may contribute to endothelial injury, vascular erosion, and delayed cardiac or vascular perforation [1,2].

No procedural complications were observed during catheter insertion in Case 1, and both central venous catheters functioned adequately following placement. However, AP chest radiographs, which are routinely performed at our institution to verify catheter tip position, demonstrated intracardiac localization. This finding was underestimated, and no corrective actions were undertaken. We hypothesize that prolonged intracardiac positioning of the catheter tip—four days in Case 1 and six days in Case 2—combined with continuous intravenous infusions, contributed to progressive mechanical erosion of the cardiac wall and subsequent catheter migration. In Case 1, additional chemical injury related to parenteral nutrition may have further contributed to endocardial damage and structural weakening.

Although pericardial disease and cardiac tamponade may arise from multiple etiologies, in these cases late catheter-related dislodgement appears the most plausible mechanism, given the persistent intracardiac position of the catheter tip over several days and the absence of recurrence after catheter removal.

## 4. Limitations and Issues of Concern

Major limitations of our report are the very small sample size, comprising only two cases, and its retrospective observational design, which preclude generalization of the findings as well as statistical analysis of risk factors. Nevertheless, in both patients, the fatal outcome was primarily attributable to an iatrogenic complication and delayed initiation of appropriate emergency resuscitative measures, despite the presence of severe underlying disease. Multiorgan failure was the result of prolonged tissue hypoperfusion and irreversible organ damage due to delayed initiation of definitive resuscitative intervention (pericardiocentesis). These events prompted a critical reassessment of our clinical practice and of potential system-related omissions in order to reduce the risk of similar complications in the future.

CVCs have been used in our intensive care unit for more than 30 years, with complications occurring infrequently and fatal events remaining exceptionally rare. Historically, most adverse events in our practice were early complications arising during catheter insertion or within the first hours after placement. In some situations, we considered positioning of the distal catheter tip within the right atrium acceptable, particularly when the catheter had already been secured to the skin with sutures or when attempts at repositioning were judged to carry a risk of catheter compromise—especially in technically challenging cases such as extremely-low-birth-weight neonates or patients with difficult vascular access.

In retrospect, we recognize that improper catheter tip positioning remained underestimated, particularly in the second case, in which chest radiographs were performed primarily to assess pulmonary pathology rather than to verify catheter position, as the catheter had been inserted in another medical institution. Insufficient experience and specific training among part of the on-call medical staff, the rarity of CT and the consequent limited clinical focus on this entity within the team, as well as the absence of significant CVC-related complications in our department in recent years, may have contributed to delayed recognition of the risk.

Additional limitations include the absence of biochemical analysis of the pericardial fluid obtained in Case 1, which could have provided stronger evidence supporting catheter-related extravasation of infused solutions. Although considered unlikely, alternative etiologies such as acute pericarditis cannot be definitively excluded. Of note, detailed information regarding the catheter specifications, insertion technique, and radiographic verification of catheter tip position in Case 2 was unavailable for review, as the catheter had been inserted at an external institution. Finally, direct visualization of the catheter tip within the pericardial space was not achieved, and post-mortem examinations were not performed due to parental refusal of consent in both cases, which precludes definitive confirmation of the mechanism of perforation.

These shortcomings prompted us to implement stricter institutional protocols for objective verification of CVC tip position using both radiographic and ultrasound assessment, and to avoid intracardiac catheter placement except in critical emergency resuscitation settings.

## 5. Conclusions

Cardiac tamponade is a rare but life-threatening complication following central venous catheter placement in pediatric patients. Post-procedural chest radiography, as well as serial control imaging, play an important role in confirming correct catheter positioning. When intracardiac placement of the catheter tip is identified, prompt withdrawal and repositioning should be performed, as this may reduce the risk of delayed catheter migration and subsequent complications. Cardiac tamponade should be suspected in any pediatric patient with a central venous catheter who develops sudden cardiocirculatory deterioration. Early clinical recognition, followed by prompt echocardiographic confirmation when available, is essential to reduce the risk of fatal outcomes.

## Figures and Tables

**Table 1:** Comparative summary detailing the two presented cases. *N/A—not available*.

Variable	Case 1	Case 2
Age	25 days	2 years
Gender	Male	Male
Diagnosis	Short-bowel syndrome	Pneumonia/Leigh syndrome
Indications for placement of CVC	Parenteral nutrition/Exhausted peripheral venous access	Intensive care management/Continuous intravenous therapy
Ultrasound guidance	No	N/A
Radiographic control after CVC placement	Yes	N/A
Type of CVC	Dual-lumen 4 Fr, 13 cm polyurethane CVC with a soft tip (Certofix^®^ Duo Paed S413, B. Braun, Melsungen, Germany)	N/A
Type and rate of infusions	Parenteral nutrition (10–15 mL/h), crystalloid solutions (8–10 mL/h), blood products (8 mL/h), anti-infective treatment (10 mL/h)	Crystalloid solutions (20–40 mL/h), blood products (30–40 mL/h), anti-infective treatment (40–50 mL/h)
Time interval from CVC placement to CT	4 days	6 days
Echocardiographic confirmation of CT	Yes	Yes
Amount of the aspirated pericardial fluid	40 mL	120 mL
Time interval from CT to clinical deterioration	3 days	12 days
Final outcome	Fatal outcome	Fatal outcome

## Data Availability

The raw data supporting the conclusions of this article will be made available by the authors on request.

## Abbreviations

The following abbreviations are used in this manuscript:

CT	Cardiac tamponade
CVC	Central venous catheter
PICU	Pediatric intensive care unit
RMB	Right main bronchus
AP	Anteroposterior
